# Development of an experimental model for radiation-induced inhibition of cranial bone regeneration

**DOI:** 10.1186/s40902-018-0173-1

**Published:** 2018-11-22

**Authors:** Hong-Moon Jung, Jeong-Eun Lee, Seoung-Jun Lee, Jung-Tae Lee, Tae-Yub Kwon, Tae-Geon Kwon

**Affiliations:** 1 Department of Oral and Maxillofacial Surgery, School of Dentistry, Kyungpook National University, 2177 Dalgubeol-daero, Jung-gu, Daegu, 41940 Republic of Korea; 2 Department of Radiologic Technology, Daegu Health College, Taejeon-Dong 15, Youngsong-Ro, Buk-Gu, Daegu, Republic of Korea; 3 Department of Radiation Oncology, School of Medicine, Kyungpook National University, 130 Dongdeok-ro, Jung-gu, Daegu, 41944 Republic of Korea; 4 Department of Radiation Oncology, Kyungpook National University Hospital, 130 Dongdeok-ro, Jung-gu, Daegu, 41944 Republic of Korea; 5 Department of Dental Materials, School of Dentistry, Kyungpook National University, 2177 Dalgubeol-daero, Jung-gu, Daegu, 41940 Republic of Korea

**Keywords:** Radiation, Bone, Regeneration, Calvaria, Defect

## Abstract

**Background:**

Radiation therapy is widely employed in the treatment of head and neck cancer. Adverse effects of therapeutic irradiation include delayed bone healing after dental extraction or impaired bone regeneration at the irradiated bony defect. Development of a reliable experimental model may be beneficial to study tissue regeneration in the irradiated field. The current study aimed to develop a relevant animal model of post-radiation cranial bone defect.

**Methods:**

A lead shielding block was designed for selective external irradiation of the mouse calvaria. Critical-size calvarial defect was created 2 weeks after the irradiation. The defect was filled with a collagen scaffold, with or without incorporation of bone morphogenetic protein 2 (BMP-2) (1 μg/ml). The non-irradiated mice treated with or without BMP-2-included scaffold served as control. Four weeks after the surgery, the specimens were harvested and the degree of bone formation was evaluated by histological and radiographical examinations.

**Results:**

BMP-2-treated scaffold yielded significant bone regeneration in the mice calvarial defects. However, a single fraction of external irradiation was observed to eliminate the bone regeneration capacity of the BMP-2-incorporated scaffold without influencing the survival of the animals.

**Conclusion:**

The current study established an efficient model for post-radiation cranial bone regeneration and can be applied for evaluating the robust bone formation system using various chemokines or agents in unfavorable, demanding radiation-related bone defect models.

## Background

Radiation therapy is widely employed in the treatment of head and neck cancer, which may be performed before or after resection of the tumor. Adverse effects of therapeutic irradiation include delayed bone healing after dental extraction or impaired bone regeneration in the resultant bony defects [1]. Radiation has different effects or varied severity on bone and soft tissue [2]. Although mineralized bone is not a radiosensitive tissue, wound healing after irradiation may be impaired due to cellular and vascular damage [3]. Irradiation causes bone marrow depression and mesenchymal cell apoptosis [4] as well as delayed wound healing, tissue inflammation, and fibrosis [5]. Radiation induces a fibroatrophic process that accompanies the significant damage caused to the vascular system and hypoxia due to reduced oxygen supply and results in complete destruction of irradiated tissue with absence of regenerative potential [6, 7].

There are limited therapeutic options for reconstruction of bone damaged by irradiation due to fibrosis, cell necrosis, and severe impairment of vascular supply. Non-vascularized free bone grafts are less effective in the irradiated field; therefore, their use is discouraged [8, 9]. Microvascular free tissue transfer has thus been considered the gold standard for bone reconstruction in the field of irradiation, as it overcomes the limitations of non-vascularized grafts [10–12]. However, the disadvantages of microvascular free tissue transfer include donor site morbidity and prolonged duration of surgery and hospitalization time. Additionally, ischemia in recipient site and radiation-induced perivascular thrombosis can decrease the success rate of bone reconstruction following the microvascular free flap technique [13, 14]. Therefore, further investigation of bone reconstruction strategy in irradiated area must be performed. Several studies have demonstrated the use of various cytokines or chemicals to enhance bone regeneration in irradiated critical-sized bone defects in experimental animals [15–18].

Development of a reliable experimental model is essential to study tissue regeneration in the irradiated field. However, external irradiation devices with linear accelerator are not readily available in experimental settings. Additionally, radiation protection and safety measures are important in experimental procedures, which are not easy to achieve. Target field-specific irradiation is imperative to prevent radiation hazard to the experimental animals. X-ray machines have been suggested to be suitable irradiation devices for small rodent experiments and may serve as an alternative to the linear accelerator [19]. Several studies have utilized X-ray machines for the purpose of irradiation [20–22]; however, creation of critical-sized cranial defect using murine model has not been well established.

The current study aimed to develop a relevant mouse model of post-radiation cranial bone defect. The current experimental setting showed the definitive inhibition of recombinant human bone morphogenetic protein-2 (rhBMP-2)-induced bone regeneration after one-time, site-specific irradiation on targeted calvarial defect.

## Methods

### Conditions for animal experiment

All animals for this research were prepared after approval by the National University Laboratory Animal Ethics Committee (KNU 2014-149). Seven-week-old female mice (C57BL/6) were utilized to evaluate the effect of radiation on BMP-2-induced bone formation. The animals were assigned randomly to test and control groups, housed in separate cages, and allowed to adapt to the experimental settings for 7 days prior to the treatment.

Radiation was delivered 2 weeks before the surgical treatment, which mimics presurgical radiation therapy before surgery [18]. Radiation therapy and surgery were performed under general anesthesia (ketamine 100 mg/kg body weight, xylazine 5 mg/kg body weight). At the end of the experiments, the animals were euthanized with CO_2_ and histological specimens were harvested for further examination.

### Manufacturing the lead shielding materials for external radiation

**Fig. 1:**
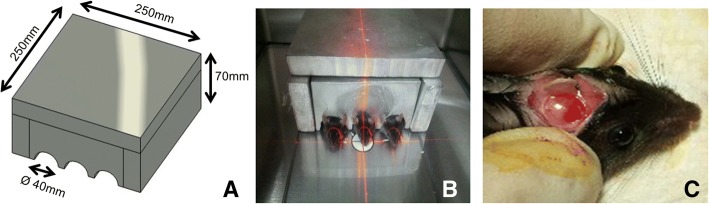
**a** Design of lead shielding block for selective irradiation to the head region. **b** Mice were adapted to a digital X-ray equipment Faxitron with radiation shield. **c** Two weeks after the irradiation, mouse calvaria defect was created. Collagen scaffold with or without rhBMP-2 was applied over the mouse calvarial defect

To localize the effect of external irradiation, a custom-made lead shielding equipment protected the rest of the body except the head. The square-shaped lead shields were manufactured as follows: molten solution of a lead alloy (over 95.5% Pb and 4 ± 0.5% Sb (antimony)) with a density of over 10.90 g/cm^3^ and a melting temperature of 70 °C (KS-A4817: ISO7212, Med-tech, USA) was poured into a Styrofoam frame to cast the shields. The thickness of the lead wall was set as 30 mm. The individually casted lead blocks were assembled as a box-shaped square shield (Fig. 1).

### Reagents and scaffold

Collagen solution (collagen: BD, USA; HEPES: Gibco®, USA; 1 N NaOH: Gibco®, USA; HBSS: Gibco® USA) was mixed with recombinant BMP-2 (1 μg/ml, PeproTech, Rocky Hill, NJ, USA) or PBS solution. After cultivating in an incubator for 12 h at 37 °C, further incubation was done at − 80 °C for 12 h in a deep freezer. Thereafter, a collagen sheet was fabricated by using a lyophilizer. Collagen sheet (width 10 mm, thickness 0.5 mm) was inserted into the calvarial defect area. Control (PBS-loaded) or BMP-2-loaded collagen scaffolds were used in experiments on cranial bone regeneration models.

Scanning electron microscopy (SEM, JSM-6700F, Jeol, Tokyo, Japan) was performed on the external surface of the collagen scaffold. Photographs were taken at a magnification of ×1,000 and ×10,000.

In vitro BMP-2 release from the BMP-2-loaded collagen scaffold (BMP-2, 1 μg/ml) was evaluated using a commercially available ELISA kit (Quantikine®, R&D systems, Minneapolis, MN, USA). A 10 mm × 10 mm-size BMP-2-loaded collagen scaffold (BMP-2, 1 μg/ml) was cut and immersed in 1 ml PBS (pH 7.4) solution. The amount of release of BMP-2 protein into the media at indicated time points was measured (*n* = 4).

### Animal experiments: radiation and surgery

Experimental animals were divided into four groups based on the presence or absence of presurgical irradiation and the application of BMP-2-loaded or empty collagen scaffold to the calvarial defect (*n* = 16, 4 animals per group): (1) control scaffold (PBS-loaded) without irradiation, (2) BMP-2-loaded scaffold without irradiation, (3) control scaffold after irradiation, and (4) BMP-2-oaded scaffold after irradiation. Under general anesthesia, the heads of the mice were irradiated with the square lead shield protecting the rest of the body.

External irradiation was performed with an X-ray equipment (Model MX 20, Faxitron X ray corporation, USA). The distance between the source and the object was set as 12 in. at 30 kvp, 0.3 mA, and 30 min. By converting the unit of Gy (gray, absorbed dose of radiation) according to the condition table supplied by the Faxitron, 4 Gy ionizing radiation was intensively delivered to the target field. Two weeks after the irradiation, the PBS- or BMP-2-loaded collagen scaffold was inserted on the critical-size calvarial defects (5 mm in diameter) in the parietal bone. Calvarial specimens that included the defect areas were harvested 4 weeks after the surgery. The specimens were stored in 4% paraformaldehyde at 4 °C for subsequent analysis.

### Radiological and histological evaluation of bone formation

To quantify new bone formation at the defect area, micro-computed tomography (μCT, Skyscan 1172, Kontich, Belgium) and histological analysis were performed. The resolution of the micro-CT was 17.09 μm voxel with 2.5 μm slice thickness, and the exposure condition was an energy of 50 kvp, current of 200 μA, and exposure time of 1.2 s. An aluminum filter (0.5 mm) was used to remove the scattered radiation. For the three-dimensional analysis of bone, the volume of interest was set as a cylindrical shape with a height of 2 mm and a diameter of 4 mm. The tomographic images were converted to Bitmap images. After reconstruction of volumetric 3D images, bone volume (mm^3^), trabecular thickness (μm), and trabecular number (1/mm) of each sample were quantified by NRecon program (Skyscan).

After the μCT analysis, the mice calvaria were decalcified for 1 week using 10% EDTA for histological analysis. The specimen was embedded in paraffin block and was sliced to a thickness of 10 μm, and trichrome and hematoxylin-eosin staining was performed. The newly formed bone areas (mm^2^) within the defect were analyzed with i-Solution software (Image & Microscope Technology, Korea).

### Statistical analysis

All analyzed data were displayed as mean ± standard deviation. ANOVA test was carried out followed by Tukey’s post hoc test using SPSS PC 10 software (SPSS Inc., Chicago, IL, USA). The level of significance was set at *p* < 0.05.

## Results

### Morphology of scaffolds

**Fig. 2:**
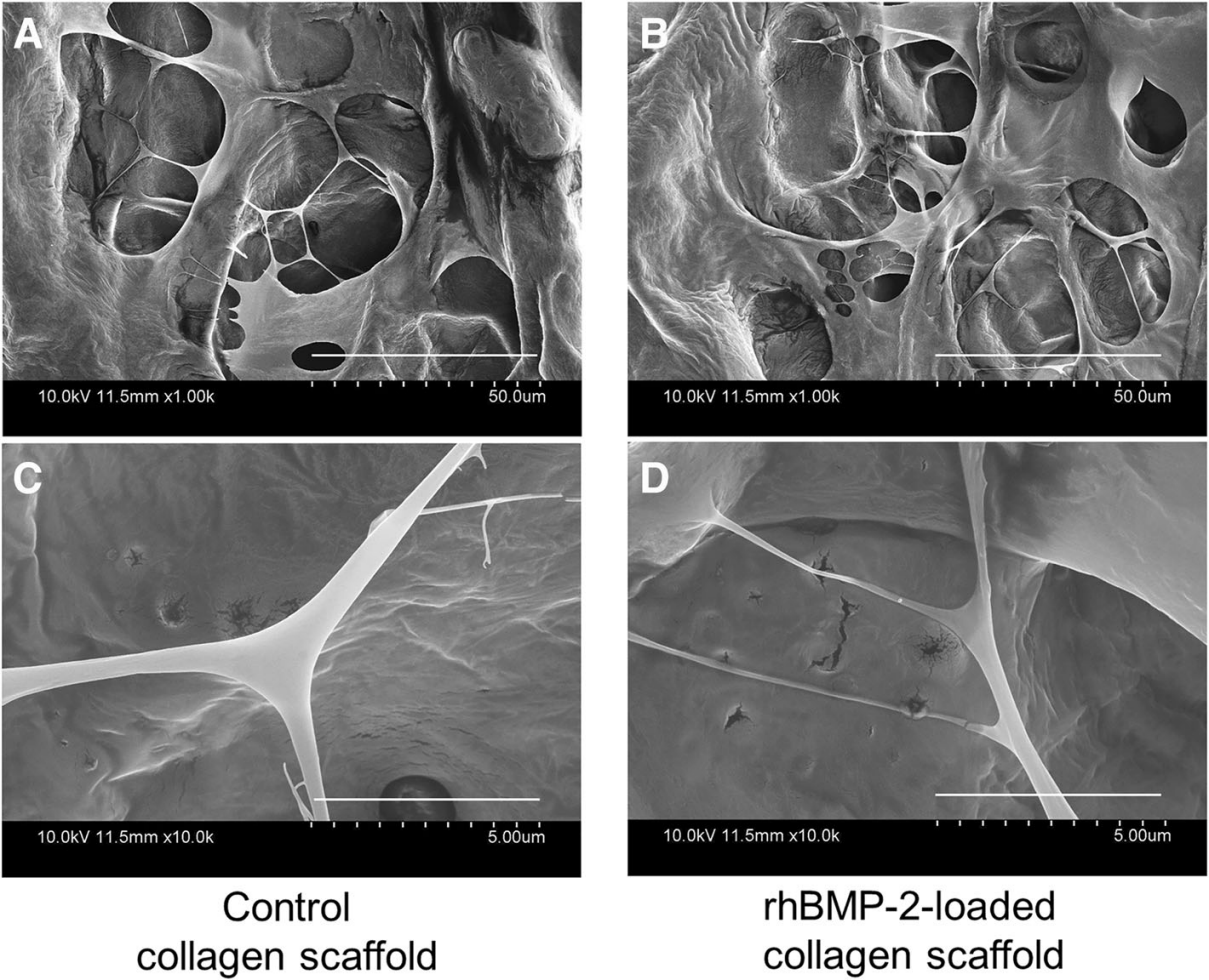
SEM images of the collagen scaffold (control, left panel, **a**, **c**) and BMP-2-loaded collagen scaffold (right panel, **b**, **d**). Images are taken at ×1000 (**a**, **b**) and ×10,000 magnification (**c**, **d**)

Figure 2 represents SEM images of the collagen scaffold (control) and BMP-2-loaded collagen scaffold. The collagen scaffold exhibited typical network structure with pores in both scaffolds. The gross structure was similar in both types of scaffolds. However, the BMP-2-loaded scaffold showed marginally higher porous structure and was separated by thinner, fibrillar networks. The BMP-2-included collagen scaffold showed more multiple layers than the control collagen sheet.

### Pattern of BMP-2 protein release from the scaffold

**Fig. 3:**
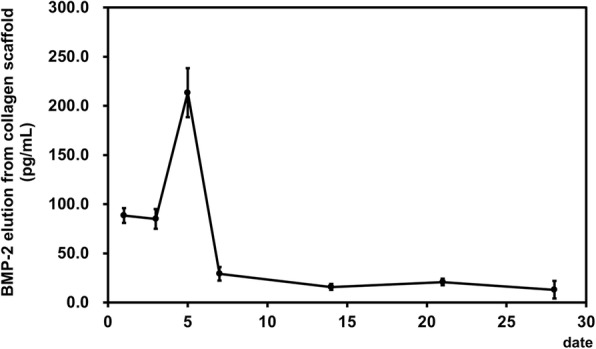
Time course of the rhBMP-2 protein secretion quantified by measuring the BMP-2 elution from BMP-2-loaded collagen scaffold

Time course of BMP-2 elution from the collagen scaffolds was investigated. On day 1, 88.5 ± 7.4 pg/ml BMP-2 was secreted and was maintained at nearly the same level on day 3 (85.1 ± 10.1 pg/ml). Significant increase was noted on day 5 (213.5 ± 24.9 pg/ml) followed by rapid decrease from day 7 (29.3 ± 6.9 pg/ml). Basal levels were maintained at the 14th, 21st, and 28th days (15.9 ± 3.0, 20.9 ± 3.3, 13.0 ± 8.9 pg/ml, respectively) (Fig. 3).

### External irradiation on calvaria and surgical treatment

All the mice maintained generally healthy condition after irradiation. Site-specific irradiation was applied to the head of mice, and there was no evidence of significant adverse events in the rest of the body. There was no sign of infection or wound rupture after irradiation and subsequent surgical implantation of scaffold into the cranial defect. Therefore, 16 samples could be harvested after completion of radiation and surgery.

### Effect of radiation on BMP-2-induced orthotopic bone formation

**Fig. 4:**
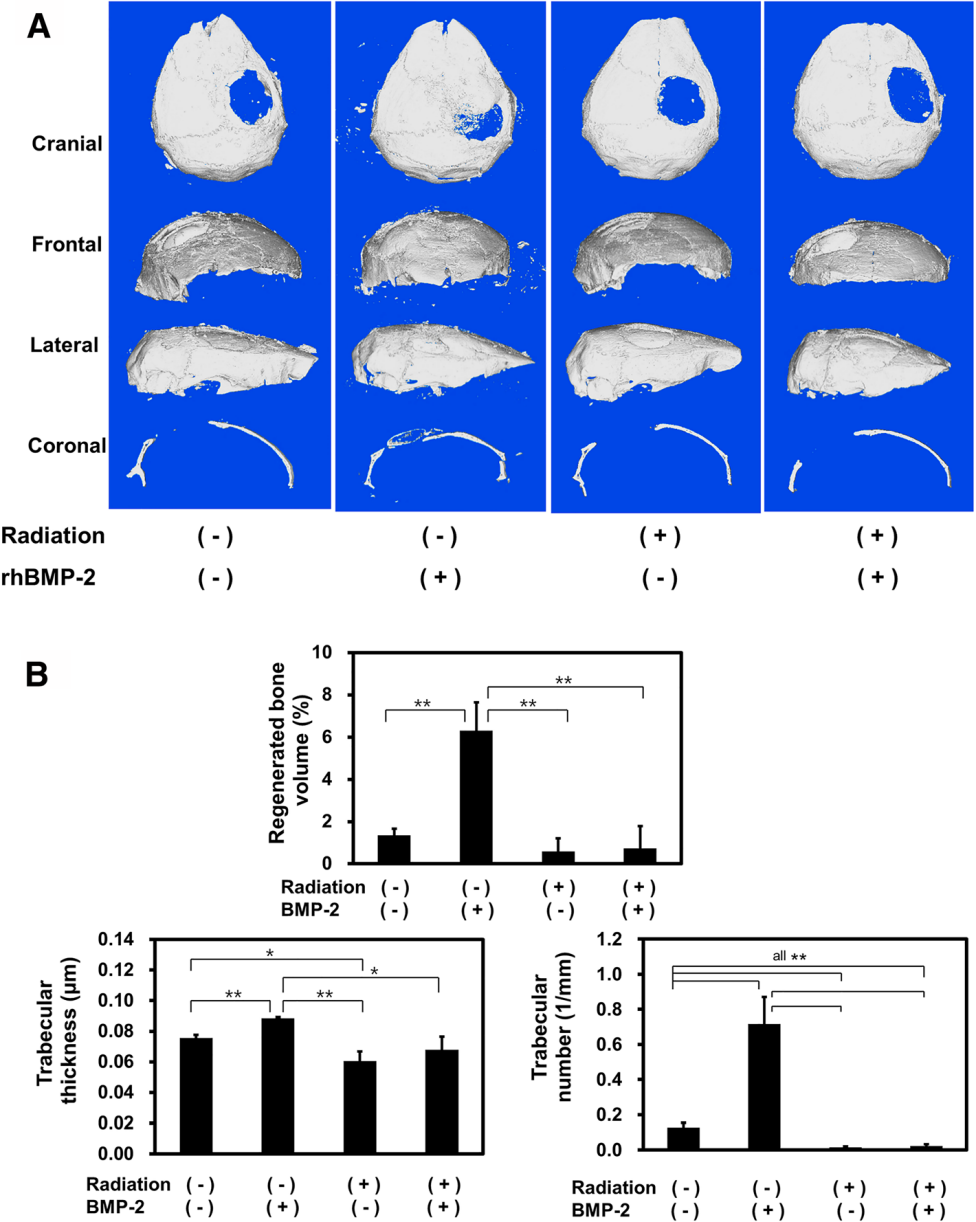
μCT evaluation of bone regeneration in the mouse calvarial defect regeneration model. **a** Each μCT image of the experimental groups. **b** Bone density analysis of the mouse calvarial defect regions for percent bone volume and trabecular thickness and trabecular number, in bone volume (%) and trabecular number. Significant inter-group differences to the other group were represented as *p < 0.05 or **p < 0.01

According to the μCT analysis of percent bone volume (mm^3^), trabecular thickness (μm), and trabecular number (1/mm), significant increase in bone regeneration was demonstrated at the non-irradiated calvarial defect reconstructed with BMP-2-loaded collagen scaffold (all *p* < 0.01). There was no significant new bone formation in irradiated calvarial defect regardless of BMP-2 treatment (*p* > 0.05). BMP-2-induced ectopic bone formation was not enhanced after irradiation, which showed that the radiation resulted in inhibition of BMP-2-mediated bone regeneration (Fig. 4).

**Fig. 5:**
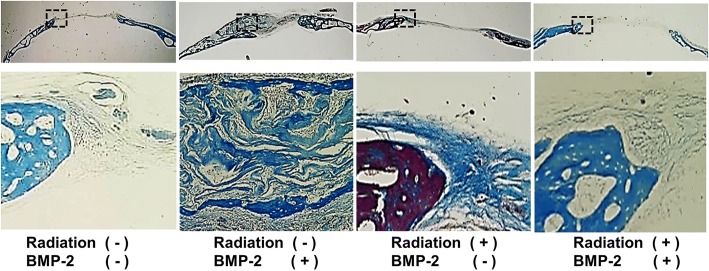
Histological findings of the calvarial specimen. Trichrome staining. Low (×1) and high magnification (×10)

**Fig. 6:**
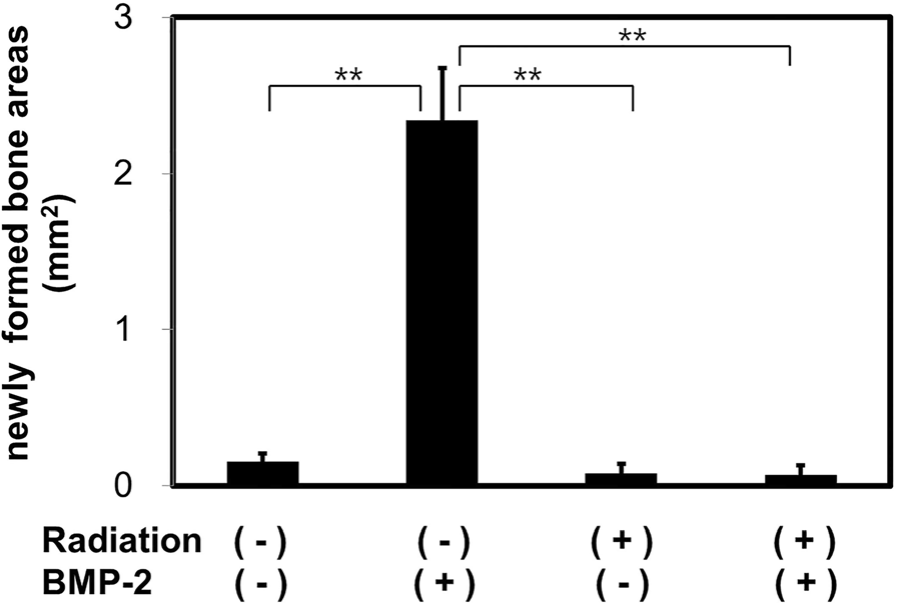
Analysis of new bone formation area from histological specimens. **Significant inter-group difference to the other group (*p* < 0.01)

Similar to the findings of radiographic density assessment, histological examination showed that BMP-2 treatment resulted in significantly more bone formation. However, radiation pretreatment resulted in significant reduction of bone formation. Histomorphometric analysis showed that bone formation at the site of the defect was significantly enhanced with rhBMP-2 (2.8 ± 0.1 mm^2^) in the control group, whereas irradiated mice showed only 0.1 ± 0.1 mm^2^ of BMP-2-induced bone regeneration (*p* < 0.05). (Figs 5 and 6).

## Discussion

Radiation therapy is widely used in cancer treatment. Although significant advances have been made, there are various adverse effects influencing the general body condition and local tissue regeneration. In humans, a single dose of 5 Gy units of radiation to the whole body can cause death and local irradiation with 5 Gy units can result in skin and hair loss and skin reddening [23]. Clinical features of acute radiation syndrome after multiple doses of irradiation include damage to the local tissues (> 1 Gy), hematopoietic system (2–6 Gy), gastrointestinal system (> 6 Gy), and cerebrovascular system (> 10 Gy) [24].

Histopathological investigations on post-radiation changes in bone structure have demonstrated that irradiation causes early inflammatory response in the cellular and vascular systems, followed by reduced vascular patency in both cortical and trabecular bones [25]. One study reported that the irradiated local mandibular region demonstrated significant reduction in vessel volume fraction and thickness [26]. According to a study on in vitro effect of irradiation on pig mandibular cortical bone, 85% of bone cell death occurred after a single fraction of 7.5 Gy [27]. Since radiation induces soft-tissue fibroatrophy and necrosis of cells, irradiated regions show varying degree of vascular insufficiency [6, 28]. Several experimental trials have been undertaken to achieve predictable and reliable bone regeneration in post-radiation defects.

Recently, various growth factors and/or cytokines have been used in animal models of post-radiation defects. Wurzler et al. used absorbable collagen sponges carrying rhBMP-2 to reconstruct 3 mm-sized rat calvarial bone defect after 2 or 7 days of irradiation (12 Gy). The group demonstrated successful results by applying a high dose of BMP-2 (25 or 35 μg) loaded in the collagen carrier [15]. Another report showed that after 20 Gy irradiation at the mandible, application of bFGF (100 μg) or rhBMP-2 (100 μg) induced bone regeneration at the irradiated site [29]. Using rhBMP-2 (0.288 mg)-incorporated collagen sponge, rabbit calvarial defects were reconstructed after 12 Gy of presurgical irradiation and showed favorable results [17]. Adenoviral gene transfer of BMP-2 has been suggested as a promising therapeutic option for reconstruction of post-irradiation calvarial defects [18]; however, there is insufficient evidence for gene therapy using BMP-7 [16]. However, the availability of adequate equipment and rodent models to develop a reconstruction strategy for this type of defect is still limited.

There is scarce literature identifying the minimum radiation dose required to exhibit the impaired post-irradiation bone regeneration in mice. One study reported that a lethal dose of radiation for 70% mice within 30 days (level of LD70/30) by total-body irradiation was 10.5–12 Gy [30]. Induction of leukemia in mice with ionizing radiation was reported at doses of 3–4.25 Gy [31]. Wernle et al. reported that 5 Gy units of local irradiation to mice hind limb could significantly change the bone morphology and strength with time [32]. An in vitro study showed that only 7.5 Gy units of radiation could significantly reduce the release of various growth factors such as VEGF, TGFß1, and BMP-2 from bone cells. They also suggested that mandibular bone cells are sensitive to low-level irradiation [27]. Considering the previously reported findings and weight of the mice, a relatively low dose of radiation was applied in this study.

Most of the previous studies used rabbit or rat model to investigate post-radiation effects on craniofacial structures. Additionally, previous studies utilized the linear accelerator that requires a special facility maintained by strict quality control. The process of transferring small animals to the large irradiator facility is cumbersome and requires general anesthesia. Small rodents do not require strong energy for irradiation. Utilizing the ordinary X-ray source for irradiation facilitates maintenance of the small animal as well as the irradiator machine during experiments. Therefore, the X-ray irradiator has been suggested for target organ-specific use or whole-body irradiation [19]. A study investigated the mean dose of scattered radiation to another animals adjacent to the radiation-targeted animal. The dose of scattered radiation was consistently within 10% [22]. In our report, lead shielding block was fabricated to protect other organs. Scattering radiation from the target (calvaria) could be minimized, and site-specific irradiation was possible under the X-ray machine. Similar to the current report, previous studies have used an X-ray machine (Faxitron) to perform experiments related to radiation-induced damage of rat groin vessels [20] or reconstruction of osseous defect of rat hind limb after irradiation [21].

The results of the present study showed that the BMP-2-treated scaffold yielded significant bone regeneration in mice calvarial defects. The BMP-2-incorporated collagen scaffold was eluted for at least 7 days. Therefore, the scaffold used in the current study could be regarded as an efficient method for binding the cytokine and scaffold for tissue engineering.

An interesting observation of this study was that a single fraction of external irradiation with soft X-ray machine eliminated the bone regeneration capacity of BMP-2-incorporated scaffold without influencing the survival of the animals. Without using the linear accelerator, preoperative radiation with an X-ray machine significantly impaired the ability of rhBMP-2 to heal critical-sized calvarial defects in mice.

## Conclusions

In conclusion, the current study established an efficient model for post-radiation cranial bone regeneration. The model can be utilized to evaluate the robust bone formation system using various cytokines or molecules in unfavorable post-radiation bone defect models. Therefore, irradiation with an X-ray machine would be a simple and suitable method to test the bone regenerative capacity of various biomaterials using small animal models.

## Abbreviations

BMP-2: Bone morphogenetic protein 2
Gy: Gray

## References

[1] Dimery IW, Hong WK (1993). Overview of combined modality therapies for head and neck cancer. J Natl Cancer Inst.

[2] Omolehinwa TT, Akintoye SO (2016). Chemical and radiation-associated jaw lesions. Dent Clin N Am.

[3] Jegoux F, Malard O, Goyenvalle E, Aguado E, Daculsi G (2010). Radiation effects on bone healing and reconstruction: interpretation of the literature. Oral Surg Oral Med Oral Pathol Oral Radiol Endod.

[4] van Os R, Thames HD, Konings AW, Down JD (1993). Radiation dose-fractionation and dose-rate relationships for long-term repopulating hemopoietic stem cells in a murine bone marrow transplant model. Radiat Res.

[5] Muller K, Meineke V (2010). Advances in the management of localized radiation injuries. Health Phys.

[6] Delanian S, Lefaix JL (2004). The radiation-induced fibroatrophic process: therapeutic perspective via the antioxidant pathway. Radiother Oncol.

[7] Kim CM, Park MH, Yun SW, Kim JW (2015). Treatment of pathologic fracture following postoperative radiation therapy: clinical study. Maxillofac Plast Reconstr Surg.

[8] Moura LB, Carvalho PH, Xavier CB, Post LK, Torriani MA, Santagata M (2016). Autogenous non-vascularized bone graft in segmental mandibular reconstruction: a systematic review. Int J Oral Maxillofac Surg.

[9] Kim JW, Hwang JH, Ahn KM (2016). Fibular flap for mandible reconstruction in osteoradionecrosis of the jaw: selection criteria of fibula flap. Maxillofac Plast Reconstr Surg.

[10] Paderno A, Piazza C, Bresciani L, Vella R, Nicolai P (2016). Microvascular head and neck reconstruction after (chemo)radiation: facts and prejudices. Curr Opin Otolaryngol Head Neck Surg.

[11] Kim MG, Lee ST, Park JY, Choi SW (2015). Reconstruction with fibular osteocutaneous free flap in patients with mandibular osteoradionecrosis. Maxillofac Plast Reconstr Surg.

[12] Pogrel MA, Podlesh S, Anthony JP, Alexander J (1997). A comparison of vascularized and nonvascularized bone grafts for reconstruction of mandibular continuity defects. J Oral Maxillofac Surg.

[13] Wang Z, Qiu W, Mendenhall WM (2003). Influence of radiation therapy on reconstructive flaps after radical resection of head and neck cancer. Int J Oral Maxillofac Surg.

[14] Beckman JA, Thakore A, Kalinowski BH, Harris JR, Creager MA (2001). Radiation therapy impairs endothelium-dependent vasodilation in humans. J Am Coll Cardiol.

[15] Wurzler KK, DeWeese TL, Sebald W, Reddi AH (1998). Radiation-induced impairment of bone healing can be overcome by recombinant human bone morphogenetic protein-2. J Craniofac Surg.

[16] Nussenbaum B, Rutherford RB, Krebsbach PH (2005). Bone regeneration in cranial defects previously treated with radiation. Laryngoscope.

[17] Kinsella CR, Macisaac ZM, Cray JJ, Smith DM, Rottgers SA, Mooney MP (2012). Novel animal model of calvarial defect: part III. Reconstruction of an irradiated wound with rhBMP-2. Plast Reconstr Surg.

[18] Hu WW, Ward BB, Wang Z, Krebsbach PH (2010). Bone regeneration in defects compromised by radiotherapy. J Dent Res.

[19] Woo M, Nordal R (2006). Commissioning and evaluation of a new commercial small rodent x-ray irradiator. Biomed Imaging Interv J.

[20] Sugiyama K, Yamaguchi M, Kuroda J, Takanashi M, Ishikawa Y, Fujii H (2009). Improvement of radiation-induced healing delay by etanercept treatment in rat arteries. Cancer Sci.

[21] Espitalier F, Vinatier C, Lerouxel E, Guicheux J, Pilet P, Moreau F (2009). A comparison between bone reconstruction following the use of mesenchymal stem cells and total bone marrow in association with calcium phosphate scaffold in irradiated bone. Biomaterials.

[22] Kirkby C, Ghasroddashti E, Kovalchuk A, Kolb B, Kovalchuk O (2013). Monte Carlo-based dose reconstruction in a rat model for scattered ionizing radiation investigations. Int J Radiat Biol.

[23] Williams JP, Brown SL, Georges GE, Hauer-Jensen M, Hill RP, Huser AK (2010). Animal models for medical countermeasures to radiation exposure. Radiat Res.

[24] Singh VK, Newman VL, Berg AN, MacVittie TJ (2015). Animal models for acute radiation syndrome drug discovery. Expert Opin Drug Discov.

[25] King MA, Casarett GW, Weber DA (1979). A study of irradiated bone: I. histopathologic and physiologic changes. J Nucl Med.

[26] Deshpande SS, Donneys A, Farberg AS, Tchanque-Fossuo CN, Felice PA, Buchman SR (2014). Quantification and characterization of radiation-induced changes to mandibular vascularity using micro-computed tomography. Ann Plast Surg.

[27] Sawada K, Fujioka-Kobayashi M, Kobayashi E, Bromme JO, Schaller B, Miron RJ (2016). In vitro effects of 0 to 120 grays of irradiation on bone viability and release of growth factors. BMC Oral Health.

[28] Doyle JW, Li YQ, Salloum A, FitzGerald TJ, Walton RL (1996). The effects of radiation on neovascularization in a rat model. Plast Reconstr Surg.

[29] Springer IN, Niehoff P, Acil Y, Marget M, Lange A, Warnke PH (2008). BMP-2 and bFGF in an irradiated bone model. J Craniomaxillofac Surg.

[30] Ryu SH, Park JH, Jeong ES, Choi SY, Ham SH, Park JI (2016). Establishment of a mouse model of 70% lethal dose by total-body irradiation. Lab Anim Res.

[31] Rivina L, Schiestl R (2012). Mouse models for efficacy testing of agents against radiation carcinogenesis - a literature review. Int J Environ Res Public Health.

[32] Wernle JD, Damron TA, Allen MJ, Mann KA (2010). Local irradiation alters bone morphology and increases bone fragility in a mouse model. J Biomech.

